# Inhibition of RAD51 by siRNA and Resveratrol Sensitizes Cancer Stem Cells Derived from HeLa Cell Cultures to Apoptosis

**DOI:** 10.1155/2018/2493869

**Published:** 2018-02-26

**Authors:** Graciela Ruíz, Heriberto A. Valencia-González, Ismael León-Galicia, Enrique García-Villa, Alejandro García-Carrancá, Patricio Gariglio

**Affiliations:** ^1^Departamento de Genética y Biología Molecular, Centro de Investigación y Estudios Avanzados, Ciudad de México, Mexico; ^2^Programa de Maestría y Doctorado en Ciencias Bioquímicas, Facultad de Química, Universidad Nacional Autónoma de México (UNAM), Ciudad de México, Mexico; ^3^Unidad de Investigación Biomédica en Cáncer, Instituto de Investigaciones Biomédicas, Universidad Nacional Autónoma de México & Instituto Nacional de Cancerología, Secretaría de Salud, Ciudad de México, Mexico

## Abstract

Cervical cancer is the second most frequent tumor type in women worldwide with cases developing clinical recurrence, metastasis, and chemoresistance. The cancer stem cells (CSC) may be implicated in tumor resistance to therapy. RESveratrol (RES), a natural compound, is an antioxidant with multiple beneficial activities. We previously determined that the expression of RAD51 is decreased by RES. The aim of our study was to examine molecular mechanism by which CSC from HeLa cultures exhibit chemoresistance. We hypothesized CSC repair more efficiently DNA breaks and that RAD51 plays an important role in this mechanism. We found that CSC, derived from cervical cancer cell lines, overexpress RAD51 and are less sensitive to Etoposide (VP16). We inhibited RAD51 in CSC-enriched cultures using RES or siRNA against RAD51 messenger RNA and observed a decrease in cell viability and induction of apoptosis when treated simultaneously with VP16. In addition, we found that inhibition of RAD51 expression using RES also sensitizes CSC to VP16 treatment. Our results suggest that resveratrol is effective to sensitize cervical CSC because of RAD51 inhibition, targeting high RAD51 expressing CD49f-positive cells, which supports the possible therapeutic application of RES as a novel agent to treat cancer.

## 1. Introduction

It is now well-known that tumors contain, within a population of nontumor-forming cancer cells, a small number of tumor-forming and self-renewing cells called cancer stem cells (CSC) [1], which have been identified in the majority of human tumors including cervical tumors and cell lines established from them [2–5]. The existence of CSC, resistant to chemotherapy, suggests that conventional chemotherapies could eliminate the bulk, but relapse may be attributed to CSC remaining unaltered, and their removal should be crucial for effective cancer therapy. Therefore, drugs that selectively target CSC, or schemes that promote their sensitization to conventional treatment, offer a greater promise for cancer therapy.

Overexpression of RAD51, a recombinase involved in DNA repair by homologous recombination (HR), is associated with a more aggressive cancer phenotype and treatment resistance in a variety of tumors, including ovarian, prostate, colorectal cancer, and malignant gliomas [6, 7]. Thus, inhibition of RAD51, directly or indirectly, will inhibit DNA repair by HR and may produce an improved response to radio- and chemotherapy treatments.

Epidemiological and dietary intervention studies in both animals and humans have suggested that diet-derived phenols, in particular flavonoids, may play a beneficial role in inhibiting, reversing, or retarding tumorigenesis in many types of cancer, including cervical cancer [8]. RESveratrol (RES) is a phytochemical polyphenolic compound naturally occurring in many plant species, including grapes, peanuts, and various herbs [9]. RES has been shown to have beneficial activities in the regulation of multiple cellular events associated with carcinogenesis. Its anticancer effects include the ability to enhance the therapeutic potential of anticancer drugs and to sensitize cancer cells to chemo- and radiotherapy [10–12]. Although these studies have examined the effect of RES on cancer, there are, to our knowledge, no studies examining the effect of RES on cancer stem cells (CSC). In the present study, we evaluate the effects of RAD51 inhibition using small interfering RNA (siRNA) or RES on CSC and found decreased cell viability and increased apoptosis, suggesting that this strategy can be used to promote the sensitization of CSC and enhance the effect of current cancer therapies.

## 2. Materials and Methods

### 2.1. Reagents and Cell Treatments

RESveratrol (RES) (trans-3,4′,5-trihydroxystilbene; >99% pure) (Sigma, 5010) was dissolved in ethanol at 80 mM and stored at −20°C; when used, it was diluted with Dulbecco's modified Eagle's medium (DMEM) to a final concentration of 137 *μ*M; Etoposide (VP16) (Sigma, 33419-42-0) stock solution was prepared at 500 mg/mL in phosphate buffer saline (PBS) and diluted with DMEM to the final concentration of 5.8 *μ*g/mL (10 *μ*M). Stock solutions of siRNA were prepared at a concentration of 1000 nM in RNAse-free water and mixed with fresh medium to a final concentration (10–30 nM).

### 2.2. Cell Cultures

The HeLa human cervical cancer cell line was obtained from the American Type Culture Collection (ATCC) and maintained as HeLa MonoLayer (ML) in DMEM supplemented with 10% fetal bovine serum (FBS) and antibiotics. HeLa SPheres (SP) enriched in CSC were cultured as previously described [4].

### 2.3. RAD51 siRNA Transfection

Knockdown of RAD51 expression was achieved using a validated siRNA silencer (Life Technologies, AM16706) targeted against exon 5 of the human *RAD51* gene. Cells were transfected for 72 h under standard culture conditions with 10 nM, 20 nM, and 30 nM RAD51 siRNA using the siPORT™ NeoFX reverse transfection reagent (Ambion, AM4510) according to the manufacturer's instructions. Following transfection, cells were replated at various densities and cultured for 48 h. Scrambled siRNA was included as control.

### 2.4. Cytotoxicity Assay

The cytotoxicity of siRNA, VP16, and RES was evaluated in cells grown in ML and SP using MTT (3-[4,5-diMethylThiazol-2-yl]-2,5-diphenylTetrazolium bromide) (Sigma, M5655). Briefly, cells were seeded in 96-well plates (5 × 10^3^ cells/well in 100 *μ*L of DMEM), incubated at 37°C overnight, and exposed to 5.8 *μ*g/mL of VP16, siRAD51 (10, 20, and 30 nM), or 137 *μ*M of RES for 48 h or for 72 h, respectively. Thereafter, 50 *μ*L of MTT (5 mg/mL) was added and incubation continued for 4 h. The medium was aspirated, and formazan was dissolved in 200 *μ*L of dimethyl sulfoxide (DMSO). Optical absorbance was measured at 570 nm using a Tecan Infinite M200 microplate reader. Experimental data was expressed as the percentage of the control group. The effect of siRNA, Etoposide, and RES on growth inhibition was assessed as percentage of cell viability; control cells (only ethanol or scrambled siRNA) were considered 100% viable.

### 2.5. Cell Cycle

Cells were collected by centrifugation, washed with cold phosphate buffered saline (PBS) solution, and fixed in 70% cold ethanol at 4°C overnight, then washed twice with cold PBS, suspended in 500 *μ*L of fluorochrome solution (50 mg/mL propidium iodide (PI), 0.1 mg/mL RNAase A and 0.1% Triton X-100 in PBS) and incubated at room temperature for 30 min in the dark. Cells were washed with cold PBS, and cycle distribution assessment was performed using a fluorescence-activated cell sorter (BD FACS Calibur). Twenty thousand events were measured per sample using flow cytometry. Cell cycle distribution was quantified utilizing cell cycle analysis software (FlowJo® 7.6).

### 2.6. Apoptosis

Apoptosis was measured using flow cytometry to quantify phosphatidylserine levels [13]. The Annexin-V FITC detection kit (BD, 556,547) was employed to differentiate apoptotic and necrotic cells. Briefly, 5 × 10^5^ cells were grown at 60% confluence and treated with different concentrations of siRAD51 (0, 10, 20, and 30 nM) or RES (137 *μ*M). Annexin-V/PI fluorescence was analyzed for each sample; the fluorescence of 20,000 cells was gated and counted using CellQuest ver. 3.3 software.

### 2.7. Western Blot

Protein concentrations were measured using a BCA protein assay kit (Pierce, 23225). Equal amounts (30 *μ*g protein) were separated by sodium dodecyl sulfate-polyacrylamide gel electrophoresis (SDS-PAGE) and transferred onto PVDF membranes, then blocked with 5% bovine serum albumin (BSA) in PBS/Tween 20 (PBST) (0.05%, *v*/*v*) for 2 h at room temperature, followed by incubation with primary antibody against RAD51 (Santa Cruz, sc-53428) at 4°C overnight. After washing with 1x phosphate buffered saline-tween (PBST) for 30 min, the membranes were incubated with secondary antibody (diluted in 5% BSA) for 1 h at room temperature. Membranes were washed three times for 15 min each with PBST. Reactive proteins were detected using a chemiluminescence kit. Data were presented as relative protein levels normalized to *β*-actin (Santa Cruz, sc-130300).

### 2.8. Comet Assay

ML or SP cells were exposed for 1 h to VP16 or vehicle (0.9% NaCl) and suspended in PBS. Subsequently, 10,000 cells were mixed with 90 *μ*L of low melting agarose (0.5% in PBS), transferred to slides precoated with agarose, and immersed in lysis buffer for 48 hours. Slides were then subjected to electrophoresis, neutralized, dehydrated with ethanol, and finally stained with ethidium bromide. The DNA fragment migration patterns of 200 cells were observed with a fluorescence microscope during 0, 1, 3, and 29 hours postexposure. The lengths of comet tails were measured from the middle of the core to the tail end as previously described [14].

### 2.9. Flow Cytometry

HeLa MonoLayer was cultured for 48 hours, and HeLa SPheres for 7 days for CD49f detection. Cells were mechanically dissociated, washed, and suspended in binding buffer. Intact cells were incubated for 45 min with primary CD49f antibody in flow buffer. Cells were washed twice in flow buffer and analyzed with an Invitrogen™ Attune™ flow cytometer blue/red lasers (Thermo Fisher Scientific). Data was analyzed with FlowJo software. CD949f antibody was coupled to fluorochrome phycoerythrin, PE (BD Bioscience, CA, USA).

### 2.10. Statistical Analysis

The results are expressed as the means ± standard deviation (SD) of a representative experiment performed in triplicate. The means were compared using the Student *t*-test assuming equal variances. *p* < 0.05 was considered statistically significant.

## 3. Results

### 3.1. Human Cervical CSC Isolated from HeLa Exhibit Chemoresistance to VP16

**Figure 1:**
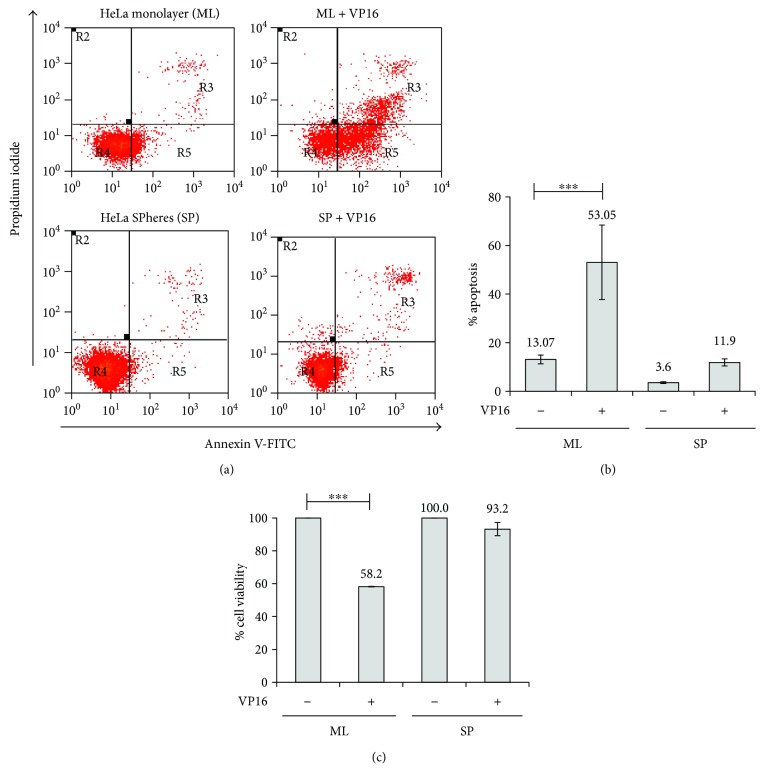
HeLa SPheres are resistant to the effects of Etoposide. HeLa SPhere and MonoLayer cultures were treated for 24 h with 5.8 *μ*g/mL of VP16. (a) Flow cytometry graphic showing Annexin-V assay in SPhere (SP) and MonoLayer (ML) cultures. (b) Graphic shows that apoptosis percentage was higher in ML cells than in SP cultures. (c) Etoposide (VP16) has no effect on SP culture viability but drastically reduces the cell viability of ML cultures. Cell viability evaluated by MTT assay (see Materials and Methods) shows that SP are more resistant to VP16 treatment than ML. ^∗∗∗^ *p* < 0.001.

We used a biological model previously developed in our laboratory [4, 5], in which HeLa MonoLayer (ML) was suspended and grown in suspension at low density in serum-free sphere medium for 7 days as HeLa SPhere (SP). These cultures are enriched in cells with similar properties to those of cancer stem cells (CSC). Now, we determined that 84.9% of the HeLa SPheres were positive for CD49f, a specific marker of CSC (Figure S1). As expected, when we evaluated the chemosensitivity of CSC from HeLa SP, we found that they were highly resistant to Etoposide (VP16) as compared to the same cells, but grown as monolayer (Figures 1(a)–1(c)). Interestingly, 5.8 *μ*g/mL of VP16 was able to induce high apoptotic levels of approximately 53% in HeLa ML cells, but only 12% in HeLa SP (Figure 1(b)). These data suggest that HeLa SP can be protected from genotoxic stress, in part by possessing a stronger DNA repair mechanism. Possibly, RAD51 may be involved in the regulation of VP16 resistance in HeLa SP.

### 3.2. HeLa SP Cultures Exhibit a More Efficient DNA Repair Mechanism When Damaged by VP16

**Figure 2:**
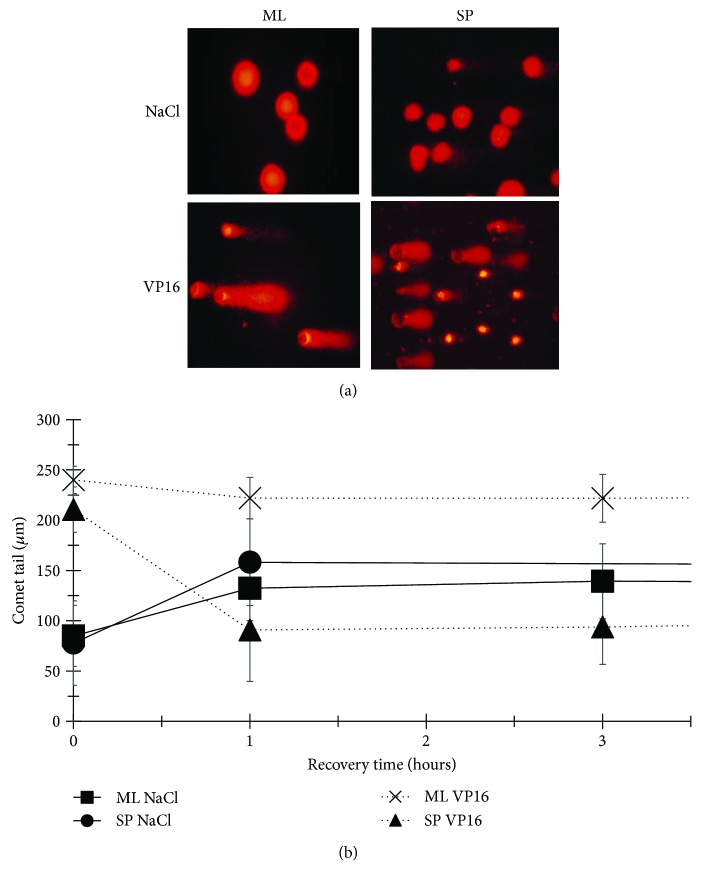
Spheres of HeLa cells repair DNA damage caused by VP16 more rapidly and efficiently than MonoLayer cells. (a) Representative fluorescence microscopy images of comet assays showing DNA damage caused by VP16 after 29 hours recovery. Short comet tail length, or its absence, indicates cells without damage; 40x objective. (b) Comet tail lengths in HeLa ML and SP within the first three hours of recovery.

Etoposide causes the fragmentation of DNA through the inhibition of Topoisomerase II, evidenced by the formation of comet-like structures. While the comet tail represents fragments of low molecular weight DNA, the head is made up of high molecular weight or nonfragmented DNA (Figure 2(a)). An ineffective DNA repair mechanism is evidenced by longer comet tail lengths that indicate greater damage and the sum of comets, the number of damaged cells. When HeLa ML were exposed to VP16, comet tails average 240 *μ*m ± 13.5 *μ*m with almost 100% of damaged cells, compared to an average of 210.53 *μ*m ± 22.56 and 100% damaged cells found for HeLa SP cultures. Importantly, while the comet tail length of HeLa ML does not decrease within 29 hours of recovery time indicating an incompetent DNA repair system, in HeLa SP, the comet tail length reduced to 93.08 *μ*m ± 37.11 within the first three hours of recovery time and the number of damaged cells dropped to 69%, suggesting that HeLa SP culture cells are able to repair the damage caused by VP16, probably because the strong expression of RAD51 (Figures 2(a) and 2(b)).

### 3.3. Knockdown of RAD51 Sensitizes HeLa SPheres to Etoposide

**Figure 3:**
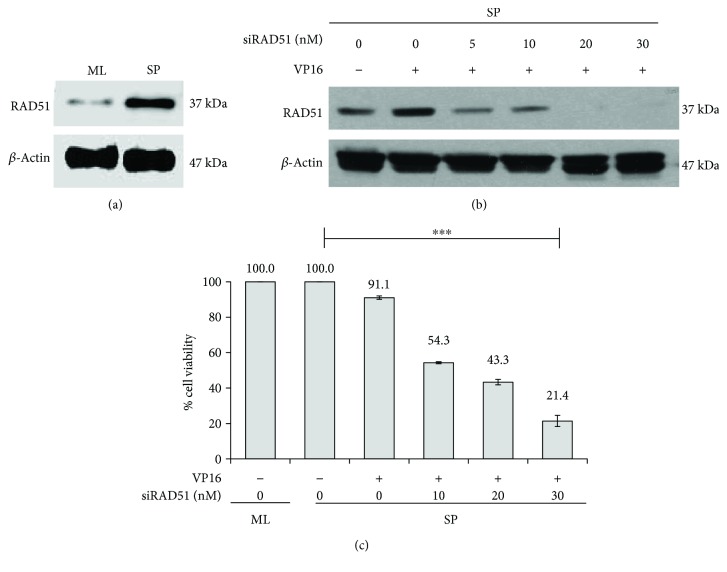
Inhibition of RAD51 decreases cell viability. (a) Western blot analysis showing RAD51 is overexpressed in HeLa SP cultures compared to the monolayer ones. (b) RAD51 expression is induced by Etoposide and efficiently inhibited by siRNA targeted to RAD51 (siRAD51). Western blot analysis shows that RAD51 expression is inhibited by increased amounts of siRAD51 (5, 10, 20, and 30 nM) in the presence of 5.8 *μ*g/mL of VP16 for 48 h. (c) Inhibition of RAD51 expression in sphere culture sensitizes to VP16. Cell viability of spheres measured by MTT decreases with increasing concentrations of siRAD51 in the presence of VP16.^∗∗∗^ *p* < 0.001.

Monolayers of HeLa cells are more sensible to Etoposide treatment and interestingly, RAD51 protein levels are lower than those exhibited by SP cultures (Figure 3(a)). To evaluate the contribution of higher RAD51 protein levels in CSC to the DNA damage response, we introduced RAD51 siRNA to HeLa ML and HeLa SP cells and treated them with VP16 for 48 h. The levels of RAD51 protein analyzed by Western blot in HeLa SP showed a decrease after treatment with 5–30 nM of siRAD51 (Figure 3(b)); however, the optimal decrease was obtained using 20 nM and 30 nM after 72 h of treatment.

When HeLa SP viability was determined, a significant effect was observed at 10–20 nM siRAD51 with 54.3 and 43.3% viability, respectively (Figure 3(c)), with 30 nM siRAD51 as the most effective treatment (21.4%). Thus, the cell viability of HeLa SP after treatment with RAD51 silencer (20, 30 nM) was much lower following VP16 (5.8 *μ*g/mL) treatment as compared to HeLa SP treated only with VP16, suggesting that knockdown of RAD51 sensitizes HeLa SP cells to VP16 (Figure 2(b)). Interestingly, this decrease in cell viability corresponds to the observed reduction in RAD51 protein levels (Figures 3(b) and 3(c)), suggesting that the cytotoxic effect of VP16 on HeLa SP was significantly increased with siRAD51 treatment.

### 3.4. RAD51 Inhibition Causes Apoptosis

**Figure 4:**
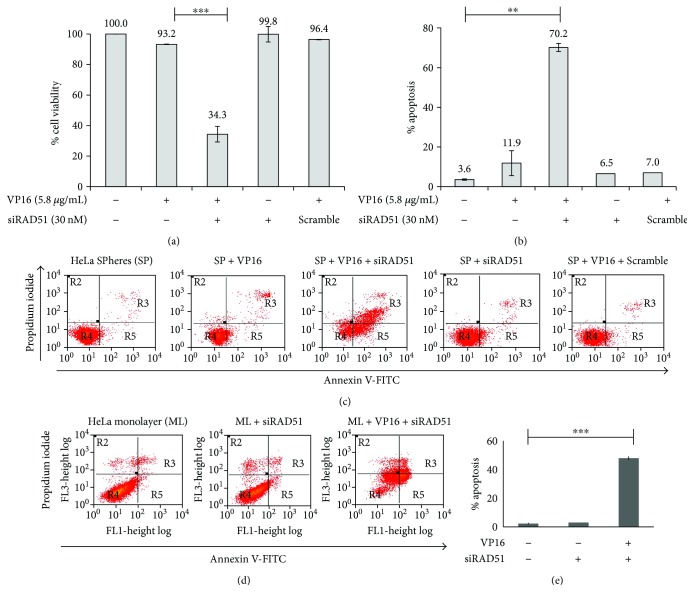
Inhibition of RAD51 expression and Etoposide induces apoptosis in sphere cultures. (a) HeLa SPhere culture was exposed to 30 nM of siRAD51 and 5.8 *μ*g/mL of VP16. By MTT assay, cell viability was affected in the presence of both compounds, but not when they were added independently. The absence of RAD51 protein decreased the cell viability of spheres exposed to VP16. HeLa SP culture exposed to VP16 and 30 nM of random siRNA (scrambled) was used as control. (b and c) Apoptosis was evaluated by Annexin-V assay (see Materials and Methods) under identical conditions; the highest level of apoptosis was exhibited in the presence of both VP16 and siRAD51. The absence of RAD51 protein sensitized spheres to VP16. (d and e) Control indicating that treatment with siRAD51 and Etoposide induces apoptosis in ML cells. ^∗∗^ *p* < 0.01 and ^∗∗∗^ *p* < 0.001.

To determine whether the beneficial effect of siRAD51 concerning the VP16-induced reduction of cell viability was related to an increase in apoptosis, we transfected HeLa SP cells with siRAD51 or a mixture of nonspecific siRNA (scrambled) and performed an Annexin-V/PI staining assay after VP16 treatment. With 30 nM of anti-RAD51, VP16 reduced cell viability to 34.3%, whereas VP16 alone decreased viability to only 93.2% (Figure 4(a)). The apoptotic rate of HeLa SP with siRAD51 treatment notably increased, from 11.9 to 70.2%, whereas there were no obvious differences in cell apoptosis in the different control groups utilized (Figures 4(b) and 4(c)). Furthermore, the decrease in cell viability and increase in apoptosis by siRAD51 (but not the scramble) indicate that these effects are RAD51-specific. Also, we observed high apoptosis levels in the presence of siRNAs and Etoposide in monolayer cultures of HeLa cells (Figures 4(d) and 4(e)).

### 3.5. Resveratrol Downregulates DNA Repair through RAD51 in HeLa SP Cultures

**Figure 5:**
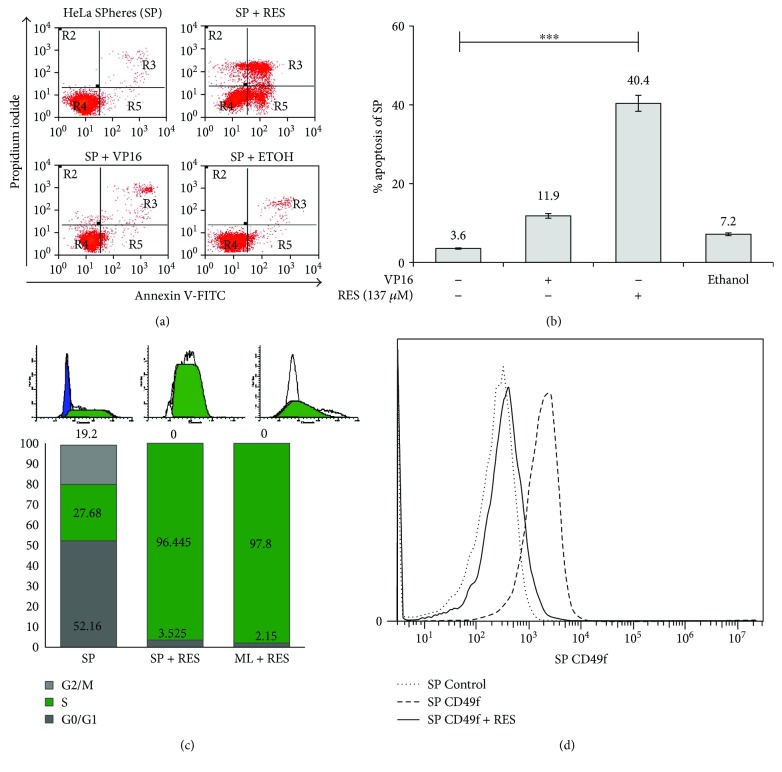
Resveratrol decreases viability and increases apoptosis of sphere cultures. (a and b) RESveratrol (RES) induces higher apoptosis levels of HeLa SPhere (SP) cultures compared to controls. Ethanol was used as vehicle. Apoptosis was evaluated by Annexin-V assay. (c) RES induced cell cycle arrest at S phase in both HeLa ML and SP cultures. HeLa cells under both conditions were treated with 137 *μ*M of RES for 72 h, and then, cells were harvested and stained with propidium iodide (PI) for 30 min at 37°C. Cells were then subjected to flow cytometric analysis to determine the cell distribution at each phase of the cell cycle. (d) Graphics show fluorescence distribution for antibody isotype (control) and CD49f-PE antibody. Histogram graphs show that CD49f-positive subpopulation decreases significantly when HeLa SP enriched in CSC are treated with resveratrol for 72 h (from 84% to 16%). The detection of CD49f cells involves the fixation and stain with CD49f-PE antibody for 45 min or isotype (see Materials and Methods). ^∗∗∗^ *p* < 0.001.

Previous work from our group suggested that RES inhibits the expression of DNA repair genes, including RAD51 [12]. Thus, we also treated HeLa SP cultures with RES. We employed flow cytometry to examine whether RES induces apoptosis in HeLa SP cultures. We observed increased levels of apoptosis (40.4%) in RES-treated HeLa SP compared with spheres treated only with VP16 (11.9%) (Figures 4(a) and 4(b)). Because it has been reported that RES can arrest cell cycle progression [15], we examined possible changes in the cell cycle of HeLa SP utilizing flow cytometry. We observed an accumulation of the cell population in S phase (96.4%) (Figure 5(c)) when spheres are treated for 48 h with 137 *μ*M RES. HeLa ML cells treated with VP16 or RES were used as control. When sphere cultures were exposed for 48 h to 137 *μ*M of RES, stained with CD49f antibody, and analyzed by flow cytometer, CD49f-positive cells decreased from 84% to 16% clearly indicating that CSC are targeted specifically by RES (Figure 5(d)). This is a new and interesting data that to our knowledge has not been previously reported.

**Figure 6:**
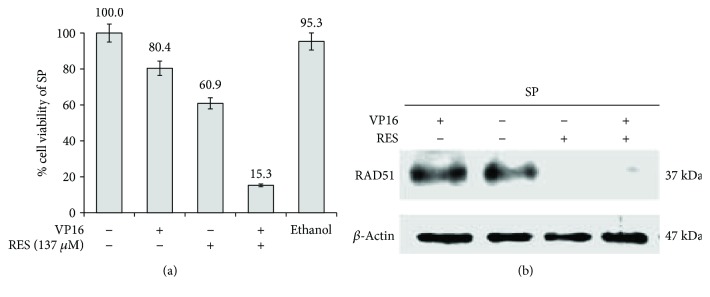
Resveratrol inhibits RAD51 expression in sphere cultures. (a) VP16 in the presence of RESveratrol (RES) decreases cell viability in HeLa SPhere (SP) cultures. Culture was exposed 72 h to both 5.8 *μ*g/mL VP16 and 137 *μ*M RES. Under these conditions, cell viability measured by MTT assay was strongly decreased (to 15.3%). Ethanol was used as vehicle control. (b) Western blot analysis shows that RES (137 *μ*M) treatment for 48 h inhibited RAD51 protein expression (similar to siRAD51) in SP cultures as compared to HeLa SP without treatment or HeLa SP treated with Etoposide. RAD51 protein levels were highly reduced when HeLa SP were treated with resveratrol alone or together with Etoposide. Untreated MonoLayer (ML) HeLa cells were also used as a control.

Then, the sphere cultures were exposed for 48 h to either 5.8 *μ*g/mL of VP16 or 137 *μ*M of RES, and cell viability was measured using MTT assays (Figure 6(a)). Under these conditions, the cell viability of HeLa SP decreased after VP16 or RES to 80.4 or 60.9%, respectively. Interestingly, the strongest reduction to 15.3% in cell viability of HeLa SP was observed when both compounds were added simultaneously. Finally, we investigated RAD51 protein levels in HeLa SP after RES treatment. As control for RAD51 expression, we treated HeLa SP with VP16 (Figure 6(b)). Interestingly, Western blot analysis showed that, in HeLa SP cells treated with RES, the RAD51 protein level is strongly decreased compared to HeLa SP without treatment, or to spheres treated with Etoposide alone (Figure 6(b)). It is worth mentioning that RES treatment affects more cancer cells than noncancer ones, since treatment of a human keratinocyte cell line (HaCaT) with RES showed that these cells are more resistant (IC50:165uM) than HeLa cells (IC50:137 uM) (not shown).

### 3.6. Proposed Model for Chemosensitivity of HeLa SPheres after RAD51 Inhibition

**Figure 7:**
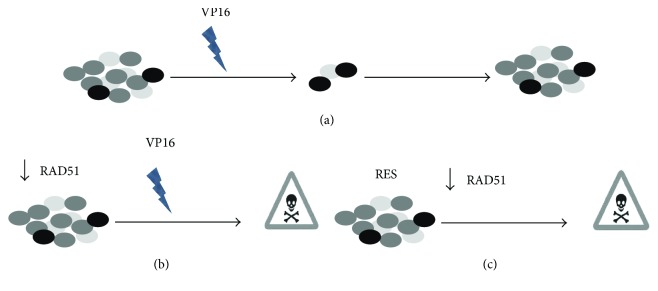
Proposed model explaining the mechanism of chemosensitization of cancer stem cells (CSC) by RESveratrol (RES) involves inhibition of RAD51. We propose that VP16-resistant CSC induced cell death (a) (as found in this work for HeLa SPhere cultures). However, when CSC are treated with siRAD51, as shown in (b), they become sensitive to VP16. Interestingly, when CSC are treated with RES, as indicated in (c), this polyphenol reduces RAD51 protein expression and induces cell death. This effect is increased in combination with VP16.

The model (Figure 7) that we propose is that CSC are resistant to VP16-induced cell death (a) (as found in this work for HeLa SP). However, when CSC are treated with siRAD51, as depicted in (b), they become sensitive to VP16. Interestingly, when CSC are treated with RES, as indicated in (c), resveratrol lowers RAD51 levels and induces cell death; this effect is increased in combination with VP16. The model is based on the observation that HeLa SPheres exhibit chemoresistance to Etoposide treatment but, after RAD51 inhibition utilizing siRNA or RES, sphere cultures become sensitive to VP16-induced apoptosis. Of major importance for this model was the observation that both RES and, in particular, RES plus VP16, increase cell death in HeLa SP cell cultures.

## 4. Discussion

Cancer stem cells (CSC) have been identified in a growing number of different types of cancer and are considered responsible for tumor progression, metastasis, therapy resistance, and subsequent tumor recurrence [16, 17]. Thus, more effective therapies require the selective targeting of this crucial cell population. We recently characterized a self-renewing subpopulation in CSC-enriched populations from four well-known human cancer-derived cell lines established from uterine cervix tumors (HeLa, SiHa, CaSki, and C-4I) [4] and found that they overexpress components of the double-strand break DNA repair machinery including RAD51 (fold change was 2.52 from HeLa SP compared with HeLa ML). Interestingly, dose-dependent radiation assays indicated that HeLa SP exhibit increased resistance to ionizing radiation [4]. Also, our laboratory established conditions to enrich CSC in sphere cultures and reported that this population expressed CD49f. In addition to this, Ortiz-Sánchez et al. reported in 2016 other phenotypic characteristics of cervical cancer stem cell-like cells; they observed increased levels of stem cell markers such as OCT-4, Nanog, or *β*-catenin in several cervical cancer-derived cell lines including HeLa [5]. Our data provide, to our knowledge, the first demonstration that inhibition of RAD51 by RES induces the chemosensitization of HeLa CSC. The results indicate that sphere cultures enriched in CSC contain a high RAD51 level and are resistant to chemotherapy with Etoposide. We hypothesized that RAD51 suppression might be a general strategy for chemosensitization of cervical CSC to the cytotoxic effects of DNA-damaging drugs such as Etoposide.

It is likely that therapeutic resistance to radiotherapy and chemotherapeutic agents could be due to a hyperactive homologous recombination (HR) capacity in tumors that overexpress RAD51 [18]. If so, lowering RAD51 may sensitize tumors to DNA-damaging treatments. We utilized siRNA against RAD51 and observed decreased cell viability as well as increased apoptosis of HeLa SP when treated with Etoposide.

Consistent with these findings, several groups have proposed RAD51 as a therapeutic target in various types of cancer, such as glioblastoma [6], pancreas [19], lung [20], colorectal [21, 22], and others, including cervical cancer [23, 24]. Depletion of RAD51 in HeLa cells by viral siRNA transfer enhances the antitumor effect of cisplatin *in vivo* [25]. More importantly, the knockdown of RAD51 in normal human fibroblasts did not increase sensitivity to cisplatin, highlighting the potential for specific targeting of RAD51 in the clinical context without adverse side effects [25]. However, our work is, to our knowledge, the first involving depletion of RAD51 in CSC from cervical cancer using RES.

Understanding how cells respond to DNA damage has facilitated the screening or rational design of agents that could selectively sensitize cells [26]. RES is a dietary chemopreventive phytochemical that has recently attracted considerable interest because of its remarkable multifunctional inhibitory effects on multistage carcinogenesis [27], including DNA repair processes. In this work, we observed that this polyphenol decreases cell viability, increases apoptosis, and induces cell cycle arrest at S phase in HeLa SP cultures. We observed decreased expression of the RAD51 protein and increased apoptosis in HeLa SP treated only with RES. Previously, we reported that *HR* genes are overexpressed in sphere cultures obtained from cervical cancer cell lines and that RES inhibits the expression of DNA repair genes such as *RAD50* and *RAD51* in the MCF-7 breast cancer cell line [4, 12].

Multiple studies have found that treatment with RES and VP16 is favorable for eliminating VP16-resistant cells through inhibition of the RAD51 protein [20, 21, 28]. We determined that RES can affect the cell subpopulation formed by CSC that was initially resistant to VP16 but that became sensitive after RES treatment, suggesting that DNA repair mechanisms involving RAD50 and RAD51 are altered. Moreover, RES inhibits invasion, migration, and the expression of proteins involved in the epithelial-mesenchymal transition (EMT) [29].

Interestingly, we found that, in HeLa SP cells, RES can induce cell death better than a well-established anticancer drug such as VP16. In this regard, apoptosis was responsible, at least in part, for RES-induced HeLa SP death. This is a possible mechanism by which RES could affect HeLa SP even better than VP16.

RES was initially recognized as a natural antioxidant [30] but, notably, recent studies examining the effects of RES on cancer cells, including CSC, have demonstrated that RES acts as a prooxidant compound, presumably in a cell type- and context-dependent manner [31], and this could explain, at least in part, its anticancer effects. In this respect, it was recently concluded that RES inhibits both the stem cell properties and viability of ovarian cancer stem cells in reactive oxygen species- (ROS-) dependent and -independent manners, respectively [32], suggesting that current information on the effects and the mechanisms of action of RES on CSC continues to be very limited [28, 29, 33–39], particularly on cervical CSC. Finally, we observed that resveratrol specifically decreases the CD49f-positive subpopulation and speculate that this effect is through regulation of specific cancer stem cell pathways, such as shown in 2013 by Sato et al., who found that resveratrol reduces the self-renewal and tumor-initiating capacity of patient-derived glioma stem cells [39].

Whereas a large body of evidence has suggested that RES inhibits the proliferation and survival of various cancer cells, there is little evidence that RES could serve as a viable treatment option once tumors are already formed [40, 41]. Therefore, our finding that RES may also decrease CSC proliferation in a DNA repair-dependent manner is encouraging. This suggests that RES inhibition of RAD51 expression in CSC leading to a decrease in cell viability and increased apoptosis may have very attractive therapeutic implications for the use of RES in cancer treatment.

## 5. Conclusions

We demonstrated that the inhibition of RAD51 expression is critical for chemosensitization of CSC, suggesting that inhibitors of RAD51, either resveratrol or siRNA, in conjunction with currently used conventional treatments, may provide a new therapeutic strategy for eliminating surviving CSC to prevent recurrence and to improve long-term survival of patients with cervical cancer.

## Abbreviations

CSC: Cancer stem cells
VP16: Etoposide
ML: HeLa MonoLayer
SP: HeLa SPheres
HR: Homologous recombination
RES: RESveratrol
ROS: Reactive oxygen species.

## Supplementary Materials

Supplementary Materials Figure S1: Spheres of HeLa cells (SP) were positive for the cancer stem cell-specific marker CD49f.
