# Supplementary material for: Inhibition of RAD51 by siRNA and Resveratrol Sensitizes Cancer Stem Cells Derived from HeLa Cell Cultures to Apoptosis
Source: Stem Cells Int. 2018 Feb 26;2018:2493869. doi: 10.1155/2018/2493869 (PMC5846439; doi:10.1155/2018/2493869)
Supplement: Supplementary Materials — Figure S1: Spheres of HeLa cells (SP) were positive for the cancer stem cell-specific marker CD49f. [file 2493869.f1.pdf]

A)

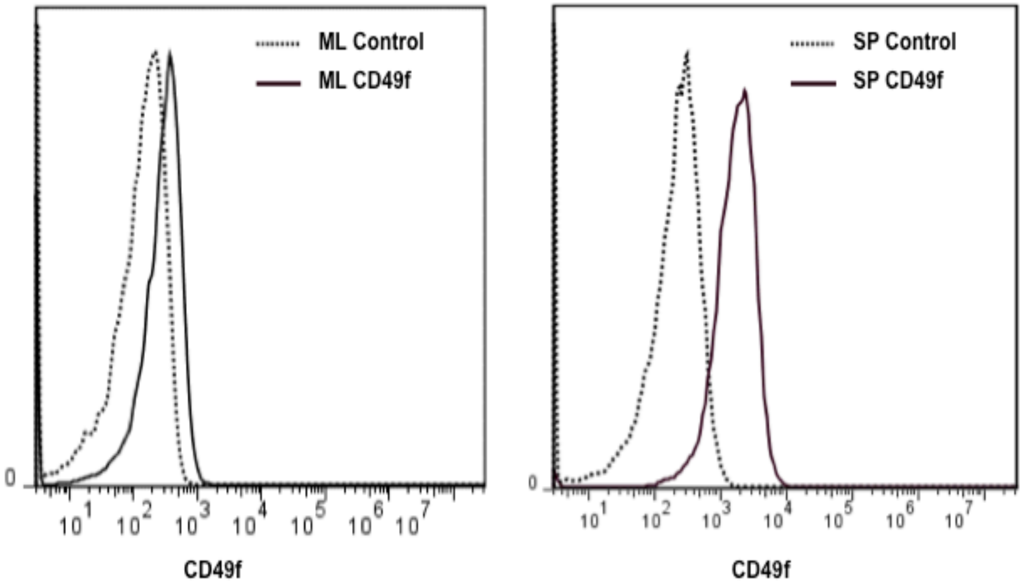

B)

Condition	Monolayer(ML)	Spheres(SP)
Isotype	0.01%	1.23%
+CD49f	0.76%	84.9%

Figure S1. Spheres of HeLa cells (SP) were positive for the cancer stem cell specific marker CD49f. HeLa monolayer (ML) cells and HeLa SP cells were cultured for 48 hours and 7 days, respectively. For detection of CD49f cells were fixed and stained with CD49f-PE antibody for 45min or isotype (see Materials and Methods). Histogram graphs show fluorescence distribution for antibody isotype (control) and CD49f-PE antibody. A) Graphics show an evident increase in fluorescence when CD49f was detected in SP cells but not in ML cells when compared to the isotype antibody. B) Table shows the percentages of CD49f cells in HeLa ML (0.76%) and HeLa SP (84.9%).
